# Insights into preventing female genital mutilation/cutting in Sri Lanka: a qualitative interpretative study

**DOI:** 10.1186/s12978-021-01114-x

**Published:** 2021-02-28

**Authors:** Angela Dawson, Kumudu Wijewardene

**Affiliations:** 1 Centre for Australian Public and Population Health Research, Faculty of Health, University of Technology Sydney, Level 8, Room 225, 235 Jones St, PO Box 123, Ultimo, NSW 2007 Australia; 2 Department of Community Medicine, Faculty of Medical Science, University of Sri Jayewardenepura, Nugegoda, Sri Lanka

**Keywords:** Female genital mutilation/cutting, Harmful practices, Prevention, Sri Lanka

## Abstract

**Background:**

FGM/C is a cultural practice associated with adverse health outcomes that involves the partial or complete removal of the external female genitalia or injury to the genitalia. FGM/C is a form of violence against women and girls. There are no laws that specifically outlaw FGM/C in Sri Lanka and no national prevalence data. There is a lack of evidence about this practice to inform prevention efforts required to achieve the Sustainable Development Goal (SDG) target 5.3.2, which focuses on the elimination of all harmful practices, including FGM/C.

**Methods:**

We undertook a qualitative interpretative study to explore the knowledge and perceptions of community members, religious leaders and professionals from the health, legal and community work sectors in five districts across Sri Lanka. We aimed to identify strategies to end this practice.

**Results:**

Two-hundred-and twenty-one people participated in focus group discussions and key informant interviews. A template analysis identified five top-level themes: Providers, procedures and associated rituals; demand and decision-making; the role of religion; perceived benefits and adverse outcomes; ways forward for prevention.

**Conclusions:**

This study delivered detailed knowledge of FGM/C related beliefs, perceptions and practitioners and provided opportunities to develop an integrated programming strategy that incorporates interventions across three levels of prevention.

## Plain English summary

Female genital mutilation, also known as female genital cutting (FGM/C), is a harmful traditional practice that can cause health problems. FGM/C is a form of violence against women and girls and is practised in many communities worldwide, including Sri Lanka. There are no laws in Sri Lanka that ban FGM/C. No government data has been collected about the number of women and girls who have been affected. There is no information about how women and girls can be best cared for and ways to prevent FGM/C in the Sri Lankan context. We undertook a study to explore community members' knowledge and views, religious leaders, nurses, doctors, lawyers, teachers, government workers, and activists in five districts across Sri Lanka. We aimed to find useful strategies to end FGM/C. Two-hundred-and twenty-one people took part in individual and group interviews. We found six main themes: Providers, procedures, and associated rituals; demand and decision-making; the role of religion; perceived benefits and adverse outcomes; ways forward for prevention. This study provides useful information about the practice of FGM/C ways to prevent it before it occurs at birth or when a woman converts to Islam. New laws, training health professionals and traditional practitioners, and educating community leaders are possible strategies to prevent FGM/C.

## Introduction

Female genital mutilation or cutting (FGM/C) also known as female circumcision, is a cultural practice associated with adverse health outcomes that involves the partial or complete removal of the external female genitalia or injury to the genitalia [1]. There are four different types of FGM/C. The most common type [1] entails the excision of all or part of the clitoris and the labia minora. The most extreme form is known as type 3 or infibulation, which entails the removal of all or part of the external genitalia and the stitching of the two cut sides, closing the vagina to varying degrees [1].

FGM/C is a form of violence against women and girls and a violation of human rights [2, 3]. The practice is associated with adverse obstetric outcomes and immediate and long-term physical, sexual and psychosocial complications resulting in injury, disability, and death [3]. The Sustainable Development Goal (SDG) target 5.3.2, adopted by all United Nations Member States in 2015 [4] focuses on the elimination of all harmful practices, including FGM/C. However, this has largely been neglected in the Asia–Pacific region [5].

FGM/C is practiced in some 30 countries of Africa, Asia, and the Middle East [3, 6], with more than 200 million children and women have undergone the procedure [3, 7, 8]. Approximately 44 million of those who have experienced FGM/C are 14 years or below. Although the incidence of FGM/C is declining in the majority of countries where it is prevalent, most of these countries are experiencing a high rate of population growth. This population growth means that the number of girls who undergo FGM/C will continue to grow if prevention efforts are not significantly scaled up [9]. Significant declines in prevalence have occurred in Africa, with the greatest reduction of 71.4 to 8.0% between 1995 and 2016 among 0–14-year-olds in East Africa. In contrast, there has been a 15.9% rise in the prevalence of FGM/C in Western Asia between 1997 and 2013 [10].

There is no nationally representative FGM/C data from Sri Lanka (UNICEF 2016a). In 2008, the Sri Lankan Ministry of Health and the World Health Organization (WHO) issued a report on violence and health in the country stating that FGM/C does “not exist in Sri Lanka” [11]. However, some have claimed that nearly 90 percent of Sri Lankan Muslims (9.5% of the Sri Lankan population, [12]) support FGM/C (Waduge 2017) and that the practice varies across different Muslim communities (Ibrahim and Tegal 2017).

There is a dearth of empirical studies on FGM/C in Sri Lanka, except a recent report by the Family Planning Association of Sri Lanka. This small qualitative study involved interviews with 26 women and 13 health providers and stakeholders in three sites to explore the practice in Sri Lanka (Ibrahim and Tegal 2019). The Media often provides a major source of information on FGM/C in Sri Lanka (Wickramage et al. 2018).

There are no laws that specifically prohibit FGM/C in Sri Lanka. However, prosecution in the case of children is possible under Section-308(A) [1] of the Penal Code (Ministry of Justice 2016). Sri Lanka has ratified several international treaties and conventions that that declare the country’s resolve to protect human rights, and specifically, to protect women and girls against violence. Sri Lanka signed and ratified the Convention on the Elimination of all Forms of Discrimination Against Women (CEDAW) (OHCHR 1979) in 1980 and 1981. In 1990, the CEDAW General Recommendation No. 14 was developed on Female Circumcision that recommended that “States parties take appropriate and effective measures with a view to eradicating the practice of female circumcision” (CEDAW 1990). Sri Lanka ratified and entered into force the International Covenant on Civil and Political Rights in 1980, which recognises the inviolability of the physical body and emphasises the importance of personal autonomy and the self-determination that human beings should have over their own bodies [13]. In July 1991, the country ratified the UN Convention on the Rights of the Child (OHCHR 1989). Article 19 of this Convention is relevant to the protection of children against FGM/C [14].

FGM/C is known as “khatna” and “sunna” in Sri Lanka; however, other local terms have been noted [15]. According to some reports, FGM/C has been clandestinely practiced in Sri Lanka for generations [16]. Supporters have disagreed with the practice being labelled as FGM/C, believing that the cut performed is not harmful [17]. Some Islamic bodies in Sri Lanka, such as the All Ceylon Jamiyyathul Ulama, which is considered the Supreme Council of Muslims in Sri Lanka, has been a strong advocate for the practice. In 2008, it issued a fatwa declaring that FGM/C is obligatory [18]. However, some Islamic religious leaders have denounced the practice [16].

Despite initially denying the existence of FGM/C, the Sri Lankan Ministry of Health issued a circular in 2018, following the report of the nation’s Parliamentary Sectoral Oversight Committee on Women and Gender, cautioning medical practitioners and authorities in the health sector against conducting FGM/C [19]. This circular was severely criticised by members of the Sri Lankan Muslim community [20]. A spokesperson from the Centre for Islamic Studies called upon the government to medicalise the practice to ensure the procedure is undertaken safely and in hygienic conditions [20, 21].

If Sri Lanka is to honour its commitment to CEDAW, the Convention of the rights of the Child and SDG target 5.3.2, then evidence based solutions to prevent FGM/C are necessary. The sensitive nature of the subject of FGM/C and the current ethnic and religious tensions in Sri Lanka highlight the need for knowledge to inform the development of activities to engage community members, health and legal professionals and policy makers in change. This paper presents the findings of the first large scale research to gain insight into the types of FGM/C practiced in Sri Lanka, individuals who perform FGM/C, the rationale for the practice and perceived effects, associated rituals and views of trends and how the practice could be prevented.

## Methods

We undertook a qualitative interpretative study to explore the knowledge and perceptions of community members, religious leaders and professionals from the health, legal and community work sectors in five districts across Sri Lanka. We aimed to identify strategies to prevent this practice. We were guided by the Standards for Reporting Qualitative Research [22].

### Setting

**Fig. 1:**
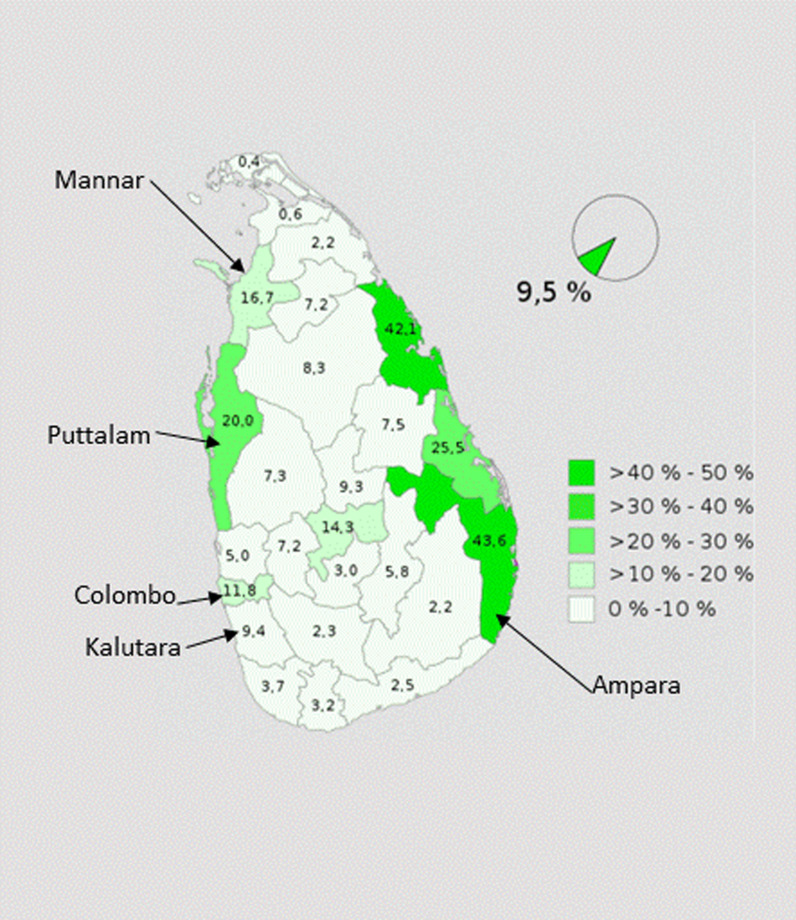
Districts where data was collected and population by religion according to districts 2012 [12]

We sought to ensure the inclusion of diverse views across communities by collecting data in the districts of Ampara, Mannar, Colombo, Kalutara and Puttalam. These areas were randomly selected from areas where considerable proportions of Muslim people reside. Colombo was selected purposively as a large proportion of the Bora community resides in this district. Figure 1 provides a summary of information on these four districts.

### Recruitment

Study participants were recruited using snowballing or chain referrals methods, a sampling approach that has been used to among minority religious groups to identify participants to study sensitive reproductive health issues [23]. Individual interview participants were recruited purposively to include public health midwives and nurses working in the labour rooms, doctors, lawyers, religious leaders, teachers, and male and female members of the community. Pregnant women were excluded from the study population, as they are a vulnerable population. Recruitment was explicitly focused on Muslim communities in the selected districts. A team leader and research assistants were employed to facilitate the recruitment of appropriate participants. These team members were of Muslim faith and knew the communities in the districts from where participants were sought. Hence, they were able to identify suitable individuals as per the inclusion criteria.

### Data collection

The views, perceptions and experiences of Muslim Sri Lankans (including Dawoodi Bohras, a sect within the Ismā'īlī branch of Shia Islam, and Muslim Malays) and non-Muslim Sri Lankans living in these communities were sought. Semi-structured key informant interviews (KIIs) and focus group discussions (FGDs) of four to seven participants were used to gather data. The team leader and five research assistants conducted the KIIs and FGDs. All researchers used the same interview guide. The second author conducted the training for members of the research team. The training sessions included information on the research objectives, proposed methodology, participant recruitment and interviewing techniques and the roles and responsibilities of the researchers. Interview questions were developed and discussed with the research team and modified based on feedback from the steering committee comprised of community members and UNFPA, UNWomen, UNICEF and UN-OHCHR in-country representatives. The interview protocol and questions were piloted in November 2017 and minor adjustments were made based on the feedback received. The interviews aimed to explore the views of professionals and community members regarding the practice of FGM/C, if and how it could be prevented.

Interviews and focus group discussions were undertaken by the field research team in the language of the participants (Tamil, Sinhala or English) using the interview guides. Interviews and FGDs lasted approximately 15–40 min, were audio-recorded and/or scribed depending on participant permission and conducted privately in locations of participants’ choice. KIIs and FDGs were conducted at the participant's place of work or at community centres. All interviews and FGDs that were not conducted in English were translated into English and back-translated to ensure accuracy. Participants were provided with a study information sheet, any questions they had were answered before written informed consent was given before the KIIs or FGDs. Researchers recruited participants until they felt assured that data saturation had been achieved. Data were analysed as it was collected and ceased when there was an agreed high level of repetition and no new data was identified in subsequent transcripts.

Participants were asked if they had heard of female circumcision, or Khatna (we used these terms when talking with participants) and if so what were the reasons it was performed, who performed, how and if there had been changes over time. Participants were also asked if it should be prevented and if so what strategies would be useful.

### Data analysis

Data were analysed using a template as per the procedure described by King [24]. The data were coded according to key categories of interest based upon the aims of the study using the qualitative software management system QSR Nvivo 12. This list of codes formed the initial template that represented the themes in the textual data. These themes were modified over time as new data revealed additional themes. For example, the reasons for the practice of FGM/C was initially comprised of two top level themes “role of religion” and “cultural motives”, but another theme, “moderation of sexual behaviour” was added as the transcripts revealed considerable data related to this factor. However, as data were analysed religion became a strong theme and cultural factors linked to this. The structure of the template, while hierarchical in nature, was also modified “Perceived health benefits and adverse outcomes” was moved further up to better articulate the relationship between participant explanations for the practice and their perceived impacts of it “moderation of sexual behaviour” became a sub-theme under the benefits. Coding to the themes was discussed by the research team and clarifications made according to feedback from the research assistants and researchers. Emerging concepts were discussed with all researchers until consensus was reached regarding their meaning and relationships with other themes. This involved establishing the top-level themes and the lower level coding associated with each theme.

## Results

A total of 221 adults (over 16 years) participated in the study. Thirty-five people 29 women and 6 men were interviewed face-to-face and 186 142 women and 44 men participated in the 19 FGDs. Information about the number of KIIs and FGDs conducted in each study district and the participants are summarised in Table 1.

**Table 1:** Number of KIIs and FGDs across selected study sites

	KIIs				FGD			
Study district	Participants	No	Age (years)	Sex	Participants	No	Age (years)	Sex
Ampara	Moulaviya	2	30, 60	F	Professionals, un/married, Muslim	12	22–37	F
	Moulvi	3	32, 35, 42	M	Un/ married, Muslim	10	19–30	F
	Osthi Mami	2	60	F	Married housewives, Muslim	12	35–60	F
					Un/ Married, Muslim	12	18–60	M
Colombo	Doctor	2	25, 37	F, M	Professionals, un/married Muslim	13	> 35	F
	Lawyer	3	58, n/a, 56	M, M, F	Married housewives, Muslim	9	35–60	F
	Civil servant	1	61	F	Professionals, un/married, Muslim	10	25–50	M
	Researcher	1	25	F				
	Professional	1	22	F				
Kalutara	Nursing Sister	2	< 45	F	Professionals, married, Muslim	3	36, 39, 60	F
Mannar	Activist	2	36, 50	F	Moulvis & business men, married Muslim	10	30–55	M
	Pre-school teacher	1	35	F	Doctor, 2 midwives, Tamil, married	3	30–55	M, F, F
	Moulaviya	2	28, 34	F	Midwives, married, Muslim	3	30–55	F
	Nurse Matron	1	60	F	Professionals, housewives married, Muslim	11	35–70	F
	Post Master	1	45	F	Professionals, un/married, Muslim	9	25–35	F
	Osthi Mami	1	70	F	Professionals, un/married, Muslim	9	25–35	F
					Married housewives, Muslim	10	18- 35	F
Puttalam	Islamic teacher	1	55	F	Professionals, married un married, Muslim	9	18- 35	F
	Midwife	4	< 50	F	Un/married, Muslim	8	18- 35	F
	Nurse	2	32, 54	F	Un/married, Muslim	10	18- 35	F
	Osthi Mami	3	50, 60, 75	F	Married housewives, Muslim	12	35–65	F
					Moulvis and business men, un/married Muslim	11	25–55	M
Total		35				186		

The participants included Osthi Mamis (traditional practitioners of FGM/C), Moulvi (male) and Moulaviya (female), Muslim religious scholars. We also interviewed an Islamic teacher working at a madrasah that provides teaching in the Koran and the Islamic faith for children 7–12 years. All KII participants were Muslim except for the Buddhist nursing sisters from Kalutara and a Doctor from Colombo and a Catholic nurse from Mannar.

The findings are described below according to the main themes from the template analysis.

### Providers, procedures and associated rituals

Older women known as Osthi Mami were described as the main providers of FGM/C. Six Osthi Mamis across three sites described receiving training from their mothers or sister.

I was trained by my mother-in-law. She taught me when I was 17. Now I am 75 years old and continue to provide khatna. I have four girls. I have taught one of my girls to do this (Osthi Mami 1, Puttalam).

Some doctors were also reported to provide FGM/C. “Doctors are also doing it secretly. They do not abide by the law; some of them do it for money.”(Ampara FGD men). However, one nurse stated, “many the doctors don’t wish to do it”. (Nurse, Mannar).

Participants described ranges of procedures.

There are ladies who do this on the clitoris. Some make a tiny incision, some cut it off, some scratch it with the nail, and some just pinch it slightly and later put medicine on it (Nurse, Mannar).

Type IV practices such as cutting, nicking, pricking, scraping and scarring were described by most Osthi Mamis, and male and female participants.

I do not cut and chop …in the secret part of the women's body there is a piece above the urinating hole, I just take that slightly. Keep the knife like this [demonstrating] – keep thumb and index fingertips together and pinch and scrape. Slightly, like a piece like a fish scale it would come and bleed slightly (Osthi Mami 2, Puttalam).

Two Osthi Mamis described the removal of flesh that may include the prepuce and clitoris that is in line with type 1 FGM/C.

There will be a little pain when it is cut and removed, and it will bleed. We should see the blood a little and cut and remove the nerve. There is a separate knife for that, called an operation knife (Osthi Mami 3, Puttalam).

It was reported that FGM/C is usually performed between seven and 40 days after birth alongside various rituals. One participant said: “they keep the child semi-submerged in the water, make the cuts and then bathe the child” (Female activist 2, Manner). Some associated shaving the baby’s hair with the practice:

On the seventh day a cow or a goat must be given as ‘akeeka’ [Islamic tradition of the sacrifice of an animal and distribute the meat on the occasion of a child's birth]. This is done for the girl child on that day. Yes, akeeka is given when they shave the hair (Moulaviya 1, Mannar).

Osthi Mamis described using a knife or blade for the procedure. “Yes, one blade to remove hair with and the other to do Khatna” (Osthi Mami, Mannar). Another participant described using “Maikkaththi [special knives] and shaving blades are brought from shop. Safety means, can do only for one or two cases. I should buy four or four. I should throw them after doing (Ampara Osthi Mami 2).

Two Osthi Mamis from Puttalam described applying cotton wool to the wound and other substances. “I apply eau de-cologne after bathing them, then it [the pain] will be reduced” (Osthi Mami 2 Puttalam). In Mannar, an Osthi Mami applied talcum powder to stop the bleeding.

The age and health status of the Osthi Mamis was seen as an issue by women and the Osthi Mamis themselves for fear that they may make a mistake and cause injury. One woman said “I was afraid as I saw her [the Osthi Mami] trembling” (FGD young women, Mannar). An Osthi Mami recalled that she “was asked not to do [FGM/C] if her hand was trembling” (Osthi Mami 2, Ampara). However, another Osthi Mami said, “There had been no such problems. Even though some say that, I have eye issue due to my age and diabetes. Most people say that even though I am old, I have never made a missed a cut, ever” (Osthi Mami, Mannar).

### Demand and decision-making

Some participants noted that FGM/C is an enduring yet clandestine practice “yes it is continuing. It is happening, though women treat it as a secret” (FGD men, Ampara). One participant recalled a recent event, “Only last week there was a new baby and she [Osthi Mami] was called in to do it.” (Older women, Colombo).

Participants spoke about the social pressure to practice FGM/C. “Educated people are against this but if their parents are culturally oriented people then they would force them to not stop this and under this pressure they would do it” (Female researcher, Colombo).

Family members were largely credited with suggesting that FGM/C be continued. This included mothers, grandmothers, mothers-in-law and sometimes men. Participant opinions varied regarding how the decision was made concerning the practice of FGM/C. For some participants this was a communal decision. “It usually a village decision. (Female activist 2, Mannar), “It is the decision of the mother and father to do this” (Nurse, Mannar). However, mothers-in-laws were reported to be very influential. “The Mother in law of my daughter was told it is compulsory.” (FGD older women, Puttalam).

Some participants noted a change in the prevalence of FGM/C: “It was 90% ten to fifteen years ago. It has been reduced due to awareness of fathers and the education level of mothers. It could be reduced to 15–20%” (Moulvi 2, Ampara). Osthi Mamis commented on the change in the demand for FGM/C and its impact on their livelihoods.

Some of them [Osthi Mamis] say that we are doing [FGM/C] for our survival. We are invited once every six months or once a year. We could be starving and lacking money until that time. We used to do [FGM/C] once a month. I am not going to accept it [the current situation] (Osthi Mami 1, Puttalam).

### The role of religion

One religious scholar interviewed in this study identified three groups with different opinions on the matter of FGM/C; “one is, who has given a fatwa on this issue, another said it is not necessary for women and then there are ones who say ‘do it if you like’” (Moulvi 1, Ampara). Those participants who described FGM/C as a “compulsory” religious obligation (FGD young women 1 and 2, Puttalam, Moulaviya 2, Ampara) also linked it to a religious custom. One participant stated, “It is because it is a Sunnah [traditional Islamic custom and practice, both social and legal, based on the verbally transmitted record] and so must be done—Like how there is Khatna for men, there should also be Khatna for women.” (FGD older women, Colombo). Others said that is approved by the Prophet Muhammad, and therefore, “It should be done. There are Hadeez [a record of statement, or action or tacit approval of Prophet Muhammad] for that” (FGD men, Puttalam). Reference was made to the fatwa justifying FGM/C made by the All Ceylon Jamiyyathul Ulama. One male participant strongly suggested that Muslims were treated as outlaws for practicing their religious customs: “It is in Islam and it should be done for children as it is mentioned in Islam. It is outsiders who make this a huge issue and try to criminalize the Muslims” (FGD men, Puttalam). Some participants regarded circumcision as a religious obligation for males but not for females, “Islam doesn’t require women to do it, but has made it a must for men” (FGD professional women Mannar). However, a Moulvi from Ampara stated that new Islamic thought does not call for FGM/C because the “Hadeez are fake”. This Moulvi and FGD participants from Puttalam and Ampara pointed to the views of other religious and medically trained leaders who called for FGM/C not to be practiced.

While some participants were certain that FGM/C was a religious obligation, they could not recall or were not aware of the rationale for performing it. Even religious leaders could not point to the exact reason: “I am not aware of any proof, but yes it is part of Islam (Female Moulaviya 1, Mannar). Osthi Mamis were also unsure of the reason for the practice but that it had been undertaken for generations. However, they continued to practice FGM/C: “They said that this wasn’t part of Islam, but they say that it is. I still keep doing this around my usual circuit” (Osthi Mami, Mannar).

FGM/C was reported to be a requirement for women who wished to marry a Muslim man and convert to Islam. An Osthi Mami described:

I did this to a woman who had embraced Islam, after teaching her the Kalima [texts to memorize to learn the fundamentals of Islam] and bathing her. Similarly, there was a girl who eloped and I did it for her too after she embraced Islam. For older women, I usually take them to V.O.G [visiting obstetrician gynaecologist] and they would instruct me on how to do it. I would ask them to shave and clean themselves and then I come and do it [the FGM/C] (Osthi Mami, Mannar).

Anther Osthi Mami stated that she performed FGM/C to facilitate conversion to Islam alongside punishment that was handed down by religious leaders because a woman had sexual intercourse before marriage:

The mosque board would instruct me to do it. Then I would perform Khatna for them. If there other complications, such as sins, then the mosque would ask me to give the appropriate punishment-hundred and one beating- to the women while the mosque board would carry out that punishment for the men. Once that is done, I would perform Khatna and then bathe her, teach her to pray and all other things in Islam. Is she is a learned girl then I would advise her to be serious about this and to increase her knowledge by reading (Osthi Mami, Mannar).

### Perceived benefits and adverse outcomes

The participants cited a number of health benefits that they believed to be associated with FGM/C. One nurse said that she had heard that FGM/C promotes sexual health and relationships saying, “They say they have good family life and sexual relationship” (Nurse 1, Puttalam). Several participants described the importance of FGM/C to moderate women’s sexual behaviour, safeguard monogamy and “prevent extramarital sex” (female Doctor, Colombo). A health professional said, “It is a ritual to keep women from not going astray; not to have affairs etc.” (FGD health professionals Mannar). An Osthi Mami stated that if FGM/C was not performed: “the child would become uncontrolled or with more sexual feeling if [the clitoris] is not taken off” (Osthi Mami 3, Puttalam).

Four participants described sexual problems, including difficulty with attaining pleasure and associated pain. One woman explained that one issue was “Taking a long time to climax and sometimes I am told women do not climax.” (Female activist, Mannar). A doctor stated that women could have “pain during sex due to exposure of nerves” (Female doctor, Colombo).

FGM/C was reported to promote cleanliness, prevent health issues that in some cases, was associated with religion. “Islam encourages this so as to avoid germs and remain healthy” (Female Moulaviya, Mannar), while others said that FGM/C “Removes some dirt which comes with us when we are born” (FGD young women, Puttalam). One woman explained that FGM/C “Can control some diseases and remove bad blood.” (Ampara FGD Professional women). An Osthi Mami stated that it “would prevent getting infected by cancer” (Osthi Mami 1, Ampara). FGM/C was also cited as “reducing all possibilities of having any urinary problems” (FGD young women, Mannar).

The health benefits were also questioned by religious leaders “I don’t think there is a benefit. There is nothing like hygiene in Khatna for women. No benefit in the hygiene and sexual satisfaction” (Moulvi 2, Ampara).

Some participants were concerned about the infections that FGM/C exposed women and babies to, “the only problem that may come is the child being infected.” (Nurse, Mannar). This, according to one woman, was due to the Osthi Mami “using unclean instruments with no training”, (FGD older women, Ampara). Two nurses from Kalutara and two midwives from Puttalam said that they had never seen any infections or obstetric issues associated with this practice. One midwife stated, “It is not a problem, there are bigger issues, like children of one group of Muslim women are not immunized because they are against it, and having home deliveries” (Midwife 3, Puttalam).

Despite this, one participant described issues with Type 1 FGM/C: “Some people take off a large part (of the clitoris) and have faced a lot health related problems and complications.” (FGD older women, Colombo). Problems were noted by another participant who said, “I recently heard of something going wrong because of an inexperienced lady doctor doing this to a girl baby” (Female researcher, Colombo).

A number of FGD participants stated that they did not believe that there were any harmful effects of FGM/C: “there is nothing to fear as it doesn’t have any negative effects” (FGD young women, Mannar).

### Ways forward for prevention

Many participants supported the prevention of FGM/C and that it should be abandoned in the future. They highlighted the importance of involving community figures and organizations in the communication of FGM/C prevention messages and inspiring change.

We should bring social change through socially accepted persons. These ideologies should be spread to the community. It would be easier for the next step. I feel that we [religious leaders] should make the first effort. We should empower like-minded people, such as Moulavis, doctors, social civil organizations, and village-level committees and spread the message through them. I think these efforts have to be made (Moulvi 2, Ampara).

Raising awareness for prevention, particularly “bottom-up” approaches were seen as a key priority: “We can stop this if we create awareness and do activities related to this, we can do it” (FGD Professional women, Ampara). Parents, students, and men were identified as important groups that should be involved in awareness-raising activities. School and workplaces were regarded as important sites for awareness-raising and the use of the Media as well as social media to transmit messages.

One nurse described the importance of women as change agents:

If Muslim women were to be made aware and decided to end [FGM/C], then it could be stopped. There are Muslim women of a higher level who should come together and raise awareness towards this matter. This is an issue for women. They can even come together with women from other communities to raise awareness about this. The women’s organizations could reach out for more support from other women and men. The support of men is important to stop this (Nurse Mannar).

Education was described as important. Several health professionals spoke about the usefulness of in-service education on FGM/C that she had received (Nurse 1, Kalutara) and one FGD participant said that they would like to receive education and a certificate on FGM/C (FGD old women, Ampara).

The need to involve religious and political leaders was noted by a number of participants because “to date no religious, political or community leaders have said FGM/C needs to be eradicated” (Female activist, Mannar). Participants cited the need for policy change as well as awareness activities: “you need to make policy decisions as well as awareness interventions from the policy level to the community level. Government involvement is not enough" (Female doctor, Colombo). A doctor said, “We need to get a few experts’ ideas and get some unbiased male religious people to say that it [FGM/C] is not required. Then it will be easy for uneducated people to justify not doing it” (Female doctor, Colombo). One Moulivaya said, “If they [the government] were to prohibit it, we should provide evidence from the Quran and Hadiths so that they [community people] can’t continue this anymore” (Female Moulivaya, Mannar).

The establishment of laws to prevent and eliminate FGM/C was also discussed. “We should we try to ban this by law” (Moulvi 2, Ampara). However, participants spoke of the need for a diplomatic and systematic approach to achieving this. One Muslim doctor said,

I think it is possible, but only with tactful measures. Bold measures such as criminalising the practise without understanding the concerns of the community could prove to be counterproductive and attract more people to this practice, which is, in fact, diminishing in my community (Male doctor 1, Colombo).

Participants also identified the need to be cognisant of the sensitivity of this subject matter and the risks taken by those who speak out against FGM/C: “A few of us who spoke against it were attacked, shamed and threatened including a few women rights activists, a couple of women politicians and a few educated men” (Female activist, Mannar).

## Discussion

FGM/C in Sri Lanka is a complex and deeply rooted socio-cultural issue that requires a multifaceted response at multiple levels. This research study has identified a number of opportunities for primordial, primary, secondary, and tertiary prevention interventions for FGM/C [25] that need to be sensitively developed in collaboration with all stakeholders and situated within the unique context of Sri Lanka. Primordial prevention comprises actions to minimize future hazards to health and therefore impedes the establishment of factors that are known to increase the risk of health issues or conditions. Primary prevention aims to prevent FGM/C before it occurs, ideally at birth or when a woman converts to Islam in preparation for marriage to a Muslim. Secondary prevention aims to reduce the impact of FGM/C by detecting the possibility of FGM/C occurring before it does. Tertiary prevention works to manage the effects of FGM/C experienced by a woman or girl. All of these strategies to prevent FGM/C should be mainstreamed [26] by integrating FGM/C-related initiatives into other sexual and reproductive programming across multiple sectors including, education, health, research, law enforcement and child protection. This ensures that FGM/C is on the agenda of the government across multiple ministries, such as those for health and education.

### Primordial prevention interventions

Participants highlighted the need for FGM related legislation to address broader health determinants aimed at the entire population rather than preventing personal exposure to risk. These strategies provide the supportive whole of society context for other prevention efforts to take effect. The establishment of specific national laws would be a necessary first step, necessitating institutions to deliver measures to prevent FGM/C [27]. Examples of legislation change could include amendments to the penal code to identify FGM/C as a specific crime or the application of child protection laws to cases of FGM/C. However, the experience of some European countries, the US, Australia and some European countries shows that it is very challenging to enforce such laws [27]. Effective prevention efforts require different sectors and appropriate professionals to be trained and engaged to facilitate a co-ordinated approach to the execution of criminal and child protection laws [28]. Significant advocacy would be required to create a specific law to render FGM/C illegal.

A group of female lawyers has, initiated work with the Ministry of Health in Sri Lanka to discuss legal solutions to prevent FGM/C [29]. This is partly in response to calls by women who have experienced FGM/C and advocates in the country [30]. However, the Centre for Islamic Studies in Sri Lanka responded by indicating their concern about the criminalisation of the practice, citing this as an infringement on their right to practice their religion [31]. This position was also reflected in our findings highlighting the importance of engaging religious leaders and reaching consensus in efforts to prevent FGM/C.

### Primary prevention interventions

Many participants noted the important role of awareness raising and education to prevent FGM/C. A national forum should be called with religious leaders in Sri Lanka to facilitate discussion and educate leaders on the harmful impact of FGM/C. Involving religious leaders in health education programmes in Ethiopia and Kenya has been found to influence communities and encourage the abandonment of FGM/C [32]. Religious leaders can act as role models who spearhead change in community behaviour and promote alternative rites and rituals to celebrate events such as the birth of a girl child, the conversion of a woman to Islam and the marriage of a woman to a Muslim man. Alternative practices could include special meals or emphasising existing rituals, such as head-shaving, bathing and religious teaching.

Media reports indicate that in recent times, alternatives approaches to FGM/C have emerged. One is known as the “butter knife method” [33]. This involves pressing a blunt knife against the abdomen and does not involve excision, piercing, or pricking of any kind. However, advocates have indicated that this disagrees with such approaches. Incentives could be provided to participate in acceptable alternative practices and vocational training provided to traditional practitioners to involve them in positive health promotion practices such breastfeeding or encouraging vaccination.

Eliminating FGM/C requires the use of diverse inter-sectoral mechanisms that have education and advocacy at their core (WHO 2016). Education efforts, as called for by the participants in this study, could be focused on building individual, professional and community awareness of FGM/C. This includes understanding FGM/C as an infringement of human rights, learning about the adverse health outcomes associated with the practice and legal status. Networks for prevention need to be built to facilitate this and involve groups such as Save the Girl Child—a group of concerned citizens representing the Muslim community in Sri Lanka, the Family Planning Association of Sri Lanka, the Mumbai-based organisation ‘Sahiyo’ and the Asia Network to End Female Genital Mutilation/Cutting [34]. Media including, social media campaigns promoting the health of girls free from violence and injury, may be useful with a particular focus on targeting prevention messages to mothers and grandmothers.

Participatory peer-to-peer educational workshops can be a useful approach to educate community members and to advocate against FGM/C. One approach in the United Kingdom involved training community champions who visit households to speak to families and provide on-going support, enabling women to speak in confidence about FGM/C issues (Mohamed et al. 2014). Educational interventions may have some effect in changing attitudes towards FGM/C and even leading to its abandonment, especially when community champions from the affected communities are actively engaged [35]. In our study, mothers, grandmothers, and mothers-in-law were cited as key to the continuation of FGM/C. Involving these women in education programs and advocacy efforts would therefore be central to changing community behaviour. Appropriate women in communities could be recruited as change agents and supported to actively empower women by promoting the value of education and their important role in decision making. This will enable women to build and use their social capital to bring about change, thereby improving the status of women, increasing autonomy and reducing gender inequity [36].

Training courses and education modules on FGM/C for the continuous professional development of health professionals should be developed. This education can prevent the medicalisation of FGM/C, improve clinician knowledge and skills in handling FGM/C survivors and be used by professionals to advocate against the practice. The Ministry of Health circular (MoH 2018) cautioning health professionals against performing FGM/C, should be accompanied by pre and in-service training for all health workers. There are examples of education modules from Australia and New Zealand [37, 38] that may provide a useful model for Sri Lanka.

### Secondary prevention interventions

Secondary prevention initiatives could include enacting specific laws to protect girls from FGM/C or integrating FGM/C into current laws. Again, considerable advocacy would be required to make such changes to legislation. Women and girls could also be safeguarded through the development of information sharing, and reporting systems could be developed so that people can report known, suspected, or at-risk cases of FGM/C to authorities in an appropriate and timely fashion. Information about FGM/C must also be integrated into the child protection training programmes of all professionals and into targeted community education programmes. FGM/C-specific assessment and intervention tools may also be useful for health and education professionals as well as for police officers and social workers [39].

### Tertiary prevention interventions

There is a need to work closely with the Sri Lankan government to promote collaboration between the health, education and justice sectors to develop accessible and appropriate support services for women affected by FGM/C. This includes the provision of support and training to health providers, counsellors and psychologists to improve the management of the medical, psychological and sexual complications resulting from FGM/C.

### Research, monitoring and evaluation of FGM/C programmes

There is no comprehensive national research programme to track the prevalence of FGM/C. More data on the prevalence of FGM/C and the needs of affected communities is required to shape the delivery of appropriate health services and prevention programmes. Future research should build knowledge of the prevalence of FGM/C through the inclusion of specific questions on FGM/C in national household health surveys, such as the Demographic Health Survey. This would provide a baseline for evaluating the effectiveness of programs and interventions. There is a need to strengthen the capacities of local NGOs and individuals to incorporate quality monitoring and evaluation into their FGM/C programming. Having a clear monitoring and evaluation framework to capture and report results can inform future FGM/C prevention initiatives. In addition, national capacity should be built to establish a system for reporting cases of suspected FGM/C so that notifications can be lodged and a response initiated. Telephone and mobile health applications could be developed as “hotlines” to enable anonymous reports from the general public and professionals.

### Limitations

Interviews were conducted in Muslim communities across five of the seven districts in Sri Lanka. The experiences and views of other communities who practice FGM/C or have discontinued this practice are not included in this study. Snowball sampling was undertaken that might have also led to gaps in the recruitment of key informants. As a result, the study may not reflect the views of all those affected by FGM/C. Despite this, there was diversity in age, gender, and profession among the participants, which included religious and community leaders.

## Conclusions

The research has identified useful insights into the practice of FGM/C in Sri Lanka and opportunities for the development of an integrated programming strategy that incorporates interventions across four levels of prevention. A comprehensive well-co-ordinated approach is therefore required, involving both bottom-up and top down primordial primary, secondary and tertiary prevention that take note of the sensitive nature of the topic.

## Data Availability

De-identified data is available upon request.

## Abbreviations

CEDAW: Convention on the Elimination of all Forms of Discrimination Against Women
FGDs: Focus group discussions
FGM/C: Female genital mutilation or cutting
KIIs: Key informant interviews
NGO: Non-Government Organisations
OHCHR: Office of the United Nations High Commissioner for Human Rights
UK: United Kingdom
UN: United Nations
US: United States
WHO: World Health Organization
